# NSAID hypersensitivity – recommendations for diagnostic work up and patient management

**DOI:** 10.1007/s40629-018-0064-0

**Published:** 2018-05-25

**Authors:** Stefan Wöhrl

**Affiliations:** 1 Floridsdorf Allergy Center (FAZ), Pius-Parsch-Platz 1/3, 1210 Vienna, Austria; 2 Department of Dermatology, Medical University of Vienna, Währinger Gürtel 18-20, 1090 Vienna, Austria

**Keywords:** Allergy, NSAID hypersensitivity, Aspirin intolerance, Analgesic intolerance, Aspirin-sensitive asthma

## Abstract

**Background:**

Adverse drug reactions (ADR) to analgesics (i.e., non-steroidal anti-inflammatory drug hypersensitivity, NSAID-HS) are one of the most common ADR, affecting approximately 1.6% of all patients. Despite the fact that they are common, they still pose a diagnostic challenge.

**Methods:**

This article is an overview of selected scientific articles and is based on research in PubMed, specialist databases, and guidelines.

**Results:**

Approximately 80% of side effects are pharmacologically predictable and are classified as type A reactions, such as abdominal pain and bleeding events. More advanced diagnostic investigations are not useful in such cases. Type B reactions, which account for the remaining 20%, are subdivided into the far more frequent cross-reactive, non-immunological NSAID-HS (acronyms NERD [NSAID exacerbated respiratory disease], NECD [NSAID exacerbated cutaneous disease], NIUA [NSAID-induced urticaria/angioedema]) and the much rarer true drug allergies of type I and IV (acronyms SNIUAA [single NSAID-induced urticara/angioedema or anaphylaxis] and SNIDR [single NSAID-induced delayed reaction]). The two latter are not cross-reactive and all other NSAIDs are generally well tolerated.

**Conclusion:**

The diagnostic work-up begins with a detailed patient’s history. Skin tests are only useful in SNIDR and SNIUAA, while in vitro tests are helpful merely in exceptional cases. In general, the diagnosis can only be confirmed by provocation testing, when required. Although cross-reactivity is usually present, provocation testing is often able to find an alternative, tolerable analgesic. Individual patient management usually enables a solution to be found for most patients.

## Introduction

“Non-steroidal anti-inflammatory drug (NSAID) intolerance” is known by many synonyms, e.g., aspirin intolerance/aspirin hypersensitivity, NSAID idiosyncrasy/NSAID hypersensitivity, as well as Widal’s disease/Samter’s triad.

**Fig. 1:**
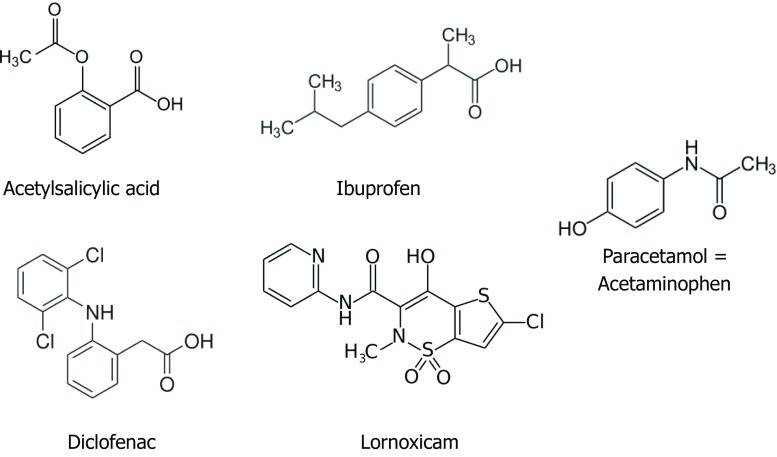
There are few chemical and structural similarities between the NSAID groups and the similarly acting paracetamol, which is also known as acetaminophen. Cross-reactivity in NERD, NECD, and NIUA (Fig. 3) is mediated via the common blockage of the COX enzyme (Fig. 2). The mechanism differs from the truly allergic SNIDR and SNIUAA where cross-reactivity is unlikely (Fig. 3)

**Fig. 2:**
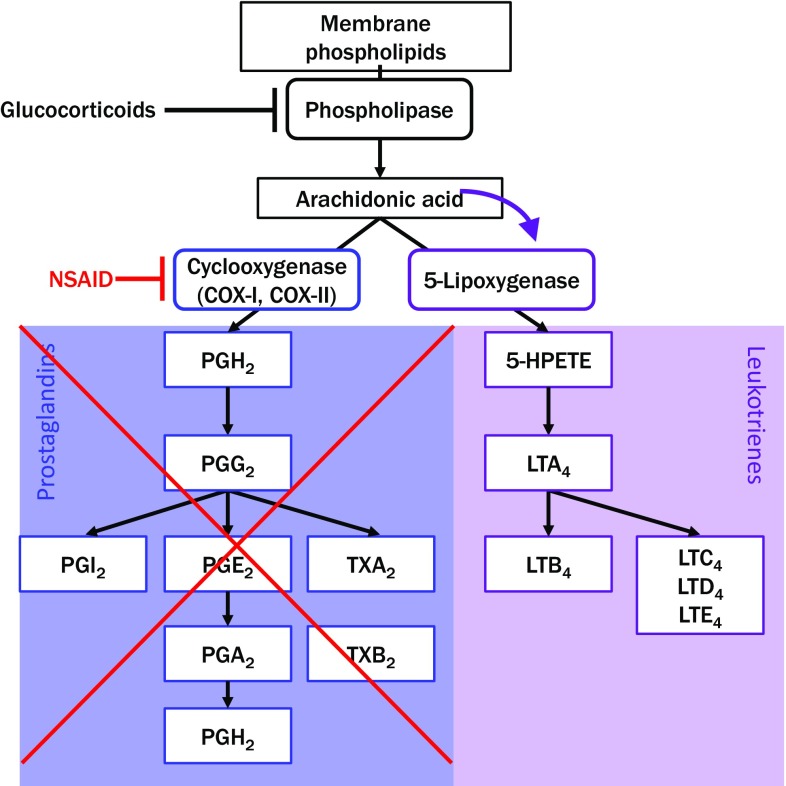
NSAIDs divert the normal arachidonic acid metabolism by blocking cyclooxygenase-1 (COX-1), which is constitutively expressed in all cells. In the case of inflammatory reactions, COX-2 may also be induced in leukocytes. Blocking COX (*red*) blocks prostaglandin synthesis (*blue*) and increases leukotriene production (*purple*), which can cause the intolerance reactions NERD (NSAID exacerbated respiratory disease), NECD (NSAID induced cutaneous disease), and NIUA (NSAID-induced urticaria/angioedema) in affected individuals (Fig. 3). (Modified from Rozsasi A and Keck T [22])

NSAIDs comprise a group of chemically diverse substances (Table 1, Fig. 1) that have one thing in common, i.e., that they inhibit the enzyme cyclooxygenase-1 (COX) and, to a lesser extent, the COX-2 enzyme. Thus, this class of drug acts on the arachidonic acid metabolism, thereby influencing the balance between leukotrienes and prostaglandins by inhibiting the production of prostanoids (Fig. 2). The definitive mechanism of NSAID hypersensitivity has not been conclusively elucidated as yet, but it is likely that NSAID-induced COX blockade results in excessive prostaglandin E_2_ production in affected individuals [1].

**Table 1:** Pharmacological classification of antipyretic non-opioid analgesics with typical examples (from Beubler E [6])^a^

Antipyretics	Class		Typical example
*Non-steroidal anti-inflammatory drug*	Salicylic acid derivatives	–	Acetylsalicylic acid
			Sulfasalazine
	Acetic acid derivatives	–	Diclofenac
			Acemetacin
			Indomethacin
	Propionic acid derivatives	–	Dexibuprofen
			Ibuprofen
			Naproxen
			Ketoprofen
			Flurbiprofen
	Enolic acid derivatives	Oxicams	Meloxicam
			Lornoxicam
			Piroxicam
		Pyrazolones	Phenylbutazone
			*Propyphenazone (historically)*
			Metamizole = dipyrone
	Fenamates		Mefenamic acid
	Selective COX-2 inhibitors		Celecoxib
*Para-aminophenol*			Paracetamol = acetaminophen

^a^Note: Most COX-2 inhibitors were withdrawn from the market because of a major pharmaceutical scandal at the beginning of this millennium in which class-specific cardiovascular side effects were downplayed in licensing trials [28]. Hence, celecoxib remains the only widely available COX-2 inhibitor

Acetylsalicylic acid (ASA; Aspirin®, Bayer and numerous generic drugs) is the best known, strongest, and only irreversible COX-1 inhibitor. Aspirin, the brand name used by Bayer, lent its name in the clinical setting in English-speaking countries for many decades and has only been replaced in recent years by NSAID (Table 1). Paracetamol, known in the Anglo-Saxon world as acetaminophen, is not a classic NSAID and its mode of action is still not completely understood. However, its side effects profile is extremely similar to that of NSAID; hence, its inclusion in this review article [2]. In addition, paracetamol is an alternative drug in NSAID hypersensitivity (NSAID-HS) that is frequently well tolerated and, in contrast to many NSAIDs, can also be used intravenously.

Adverse drug reactions (ADR) occur in 1.6% of all users following NSAID administration [3]. Most NSAIDs are not subject to medical prescription, explaining why often substantial amounts of NSAID are used, sometimes without medical monitoring. The majority (around 80%) of ADR to NSAID are pharmacological and predictable (type A), which does not necessarily imply that all of these reactions are harmless, i.e., gastrointestinal bleeding and NSAID-induced nephrotoxicity are the most frequent causes of death due to ADR to drugs [4]. In the case of chronic use, all NSAID, with the exception of salicylic acid derivatives, may pose a cardiovascular risk, most particularly COX-2 inhibitors [5]. Although hepatoxicity is feared with paracetamol use, this only occurs at high doses [6]. Type A ADR should not be submitted to any further testing because all tests will yield a predictable negative outcome.

In contrast, NSAID-HS is an unpredictable “bizarre” ADR (type B) caused by individual factors and is responsible for around 20% of ADR to NSAID [7, 8]. The rest of this manuscript will exclusively address type B reactions.

## Clinical symptoms

**Fig. 3:**
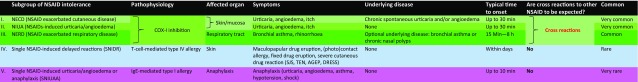
The five reaction patterns of type B adverse drug reactions to NSAID: three NSAID hypersensitivities caused by COX-1 inhibition (NERD, NIUA, and NECD) and two true allergic reaction patterns (SNIDR and SNIUAA)

In its original sense, “NSAID hypersensitivity” referred to the triad of the symptoms nasal polyps, bronchial asthma, and NSAID hypersensitivity, which is also known as Widal’s disease Samter triad. According to the more recent nomenclature, five different entities fall under “NSAID hypersensitivity” [9, 10]. The three true NSAID hypersensitivity reactions are non-immunologically mediated, cross-reactive hypersensitivities caused by changes in the arachidonic acid metabolism. It is important for clinical understanding that marked cross-reactivities between structurally widely differing NSAIDs should be expected in these three intolerances (Fig. 1), since the common basis is the blockade of the COX-1 enzyme. Some authors refer to this type of reaction as a “pseudo-allergy” [8]. There are three entities:

- Patients with skin reactions such as urticaria and/or angioedema are classified into two subgroups (see also Fig. 3):
  - Affected individuals with underlying chronic urticaria and/or angioedema where NSAID use causes an exacerbation of the underlying disease are classified as suffering from NSAID-exacerbated cutaneous disease (NECD).
  - Affected individuals without underlying chronic urticaria/angioedema are referred to as suffering from NSAID-induced urticaria/angioedema (NIUA). What is interesting here is that many of these patients to develop chronic urticaria later on; thus, NSAID use can “unmask” the subsequent onset of urticaria somewhere in the future. This resembles gestational diabetes, which often precedes true type II diabetes in the expecting mother by many years and will still be reversible following pregnancy.
- Patients with respiratory symptoms fall into one group:
  - This symptom is referred to as NSAID-exacerbated respiratory disease (NERD), formerly also known as aspirin-exacerbated respiratory disease (AERD). Clinical symptoms include rhinorrhea, blocked nose, and bronchial asthma. Many of these patients exhibit nasal polyps, chronic rhinosinusitis, and/or bronchial asthma as underlying diseases.

**Fig. 4:**
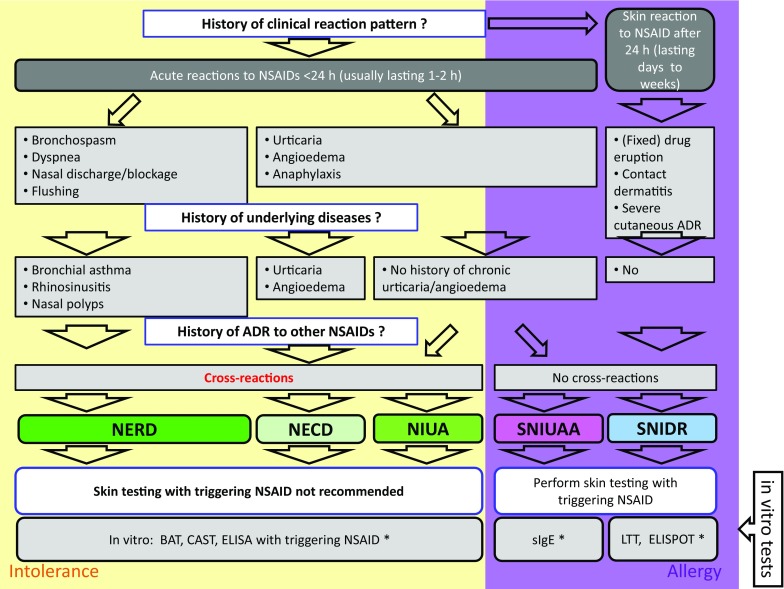
Diagnostic and management algorithm for type B adverse drug reactions (ADR) to non-steroidal anti-inflammatory drugs (NSAID); figure modified from Kowalski M et al. ([10]; also see Fig. 3. The NSAID hypersensitivity NERD (NSAID exacerbated respiratory disease), NECD (NSAID exacerbated cutaneous disease), and NIUA (NSAID-induced urticaria/angioedema), which are caused by cyclooxygenase-1 inhibition, displayed on a *light yellow* background, while the IgE-mediated type I allergy SNIUAA (single NSAID induced urticaria/angioedema or anaphylaxis) and the type IV allergy SNIDR (single NSAID induced delayed reaction) appear on a *purple* background. *None of the in vitro tests for ADR to NSAID are validated and are therefore not currently recommended for routine use [7, 10, 21]

In contrast to the earlier understanding of the term, and probably confusingly for most non-experts, the new nomenclature also embraces the much rarer, true drug allergies to NSAID for the first time ([10]; Figs. 3 and 4). Since these are true immunological reactions, immunological cross-reactions with other NSAIDs are unlikely, in contrast to the true NSAID hypersensitivity reactions (NECD/NIUA/NERD; see above)

- In rare cases, true T‑cell-mediated, delayed type IV allergic drug eruptions may occur (single NSAID-induced delayed reactions, SNIDR). As with all type IV-mediated drug allergies, disease patterns vary widely. The clinical pictures most frequently seen include clinically mild maculopapular drug eruptions (MDE), in particular to systemically administered diclofenac and metamizole, as well as mild contact allergies, again mild, to topical NSAIDs such as diclofenac and bufexamac, the latter having since withdrawn from the market for this reason [11]. Fixed MDE, in particular to oral mefenamic acid [12], are also observed, as are rare cases of life-threatening severe cutaneous drug reactions, particularly to systemically administered oxicams ([13]; Stevens-Johnson syndrome, SJS; toxic epidermal necrolysis, TEN; acute generalized exanthematous pustulosis, AGEP; and drug rash with eosinophilia and systemic symptoms, DRESS).
- True IgE-mediated type I allergic reactions (single NSAID-induced urticaria/angioedema or anaphylaxis, SNIUAA) are the rarest reactions. Propyphenazone, a phenazone derivative, used to be notorious in this regard [14] and, as a result, has virtually disappeared from the market. Another known trigger is the pyrazolone metamizole [15], which is commonly used particularly in Austria. There have been extremely rare reports from Spain of individual reactions to the acetic acid derivative diclofenac and, in particular, to the propionic acid derivatives ibuprofen, ketoprofen, and naproxen (Table 1).

## Diagnostic allergy testing

Investigating ADR triggered by NSAIDs is often unsatisfactory for the allergist. This is due to the fact that both true NSAID hypersensitivity (NECD, NIUA, and NERD) and the most important differential diagnosis of acute/chronic urticaria/angioedema are very common. As such, the most important tool in allergy diagnosis, the patient history, is often non-specific. At the same time, NSAID is a class of drugs that are particularly important in the primary care sector and for which there are a multitude of indications. Therefore, they belong to the drugs, besides antibiotics and local anesthetics, for which the international ADR consensus recommends diagnostic testing in all cases [16]. Due to the complexity of the task, testing should be performed at a center experienced in managing these types of patients [7].

As always in allergology, taking the specific medical history is the most important diagnostic factor. Particular attention should be paid to the following details (Figs. 3 and 4):

1. Which type of reaction pattern?
  - Skin-type (pruritus, flushing, urticaria, angioedema)
  - Respiratory-type (cough, bronchial asthma, respiratory distress, rhinorrhea, sneezing, blocked nose)
  - True anaphylaxis (severe drop in blood pressure, loss of consciousness, resuscitation, acute and severe gastrointestinal symptoms (i.e., >5 times diarrhea), other emergency medical measures)
2. How many reported episodes?
  - Is there evidence of cross-reactivity to other NSAID?
    What was the triggering dose? (Patients with NERD react to significantly lower doses than do patients with NECD/NIUA, e.g., 30 mg oral ASA in NERD compared with 500 mg in NECD/NIUA)
  - From a frequency of three or more episodes to different NSAIDs with the same symptoms, the likelihood of NSAID hypersensitivity increases to the extent that the diagnosis may be based solely on the basis of patient history [10].
3. Are there any characteristic underlying diseases?
  - Chronic urticaria/angioedema
    Is there evidence that oral antihistamines are able to effectively suppress the reaction?
  - Bronchial asthma
    Existing asthma medication/current lung function?
  - Nasal polyps
    i. Current nasal status?
    ii. Polypectomy in childhood? Recurrence?
  - Chronic rhinosinusitis
4. Are there any differential diagnoses that could better explain the symptoms?
  - For instance, acute, infection-related urticaria and angioedema?

### Classic NSAID hypersensitivity (NECD, NIUA, and NERD)

Since COX-1 inhibition is the mechanism of the much more common NSAID hypersensitivity (NECD, NIUA, and NERD), classic skin allergy tests remain negative and are thus not recommended ([10]; Fig. 4).

Therefore, if it is not possible to unequivocally establish the diagnosis on the basis of patient history, only provocation testing makes sense. At the same time, this offers the possibility to test patients’ tolerance of safe alternative drugs (see the section “Safe alternative drugs”). This also enables—where desired—the initiation of ASA desensitization, as has recently become fashionable, primarily as recurrence prevention in nasal polyps ([17]; see section “Miscellaneous”).

The aim of provocation testing should be to unequivocally answer one question: Can the suspected trigger really elicit the intolerance reaction? To achieve this, the suspected drug is administered (preferentially) orally in increasing single doses over the course of a day until a normal daily dose is reached [7]. Particularly in the case of ASA, this dose depends on the indication. At 50–100 mg, the cardiovascular target dose for the secondary prevention of thromboembolic events is much lower compared with antipyretic or anti-inflammatory target doses of 1000–2000 mg, e.g., in headache or rheumatic disorders.

The details involved in the planning of provocation tests go beyond the scope of this review article. Numerous different provocation protocols (oral, more rarely nasal, pulmonary, and intravenous) have been published. These often take into consideration different clinical pictures and underlying diseases, but also include a generous measure of pragmatism (e.g., national availability of approved NSAID preparations and accordingly selected dosages). The dosage for the ASA oral provocation test published in the 2007 position paper by the European Academy of Allergy and Clinical Immunology (EAACI; [18]) failed to become established in clinical routine—according to the author’s personal experience in German-speaking countries—due to the fact that different standard ASA dosages are usual there compared with the rest of Europe. For example, a possible dose increase in the case of a reaction would be 50–250–500 (–1000) mg ASA every 2 h, for other NSAIDs 1/10, 1/2, and 1/1 of the normal single dose. In the case of severe reactions, lower initial and, where necessary, intermediate doses need to be used. In this connection, the reader is referred to Bettina Wedi’s recent review article on NSAID hypersensitivity in this journal [19].

However, although provocation tests are important in the diagnosis of ADR in general and NSAID hypersensitivity in particular, resources for provocation testing are limited. NSAID hypersensitivity is a common disease with a prevalence of up to 2% in the general population, with a much higher prevalence in high-risk populations, e.g., asthma, nasal polyps, or urticaria [20]. If there are insufficient resources to perform provocation tests in all patients, it is the task of the allergist to define “high-need” patients and submit them to provocation testing in a selected manner. For example, the EAACI position paper offers the option to clinically diagnose NSAID hypersensitivity without the necessity of additional provocation testing in patients with ≥ three episodes to three different NSAIDs with similar reaction patterns (e.g., three independent episodes of urticaria for 1 h, each within 2 h of oral ingestion of 75 mg diclofenac, 500 mg ASA, and 400 mg dexibuprofen; [10]).

Some laboratories offer leukotriene measurement following in vitro stimulation of leukocyte fractions with NSAID (e.g., CAST-Elisa®, Bühlmann Laboratories, Schönenbuch, Switzerland). However, despite decades of use and optimization, this method remains insufficiently reliable to be recommended for routine use [7, 21, 22]. Similarly, the basophil activation test (BAT; the determination of activation markers following in vitro stimulation of leukocyte fractions of blood, e.g., CD63 and/or CD203c) is generally regarded as unsuitable for routine use, in contrast to IgE-mediated drug allergies [21].

### NSAID allergy (SNIDR and SNIUAA)

Classic diagnostic allergy testing using skin tests (skin prick test, intradermal test, and patch test) is only useful in the rare type IV (SNIDR) and type I (SNIUAA) allergic reactions [10]. Since these are uncommon (in the author’s experience, around 3% of referrals for NSAID hypersensitivity to his institution, unpublished data), skin testing is only helpful if the patient history points to SNIUAA (e.g., two episodes of urticaria and angioedema following 250 mg of oral metamizole for a maximum of 6 h each without recurrence) or SNIDR (e.g., MDE on day 6 of 75-mg oral diclofenac twice daily, with a resolution after stopping diclofenac only after weeks). Pyrazolones such as metamizole are the most frequent triggers of SNIUAA and, in the case of a relevant patient history, should be tested on the skin. Testing with undiluted solutions (or crushed tablets when unavailable) is currently recommended for skin prick testing at a concentration of 0.1 mg/ml in physiological saline solution for intradermal testing and 10% in vaseline for patch testing. The reader is referred to the position paper published by the European Network on Drug Allergy (ENDA) for detailed information on performing skin tests with NSAID [23].

Unfortunately, commercial, validated in vitro tests to determine IgE against NSAIDs are currently not available. From an historical perspective, the successful determination of IgE against propyphenazone using no-longer-available non-commercial ELISAs was a rare exception [14]. Although BATs are offered by a handful of laboratories and can be helpful in some cases, they are not sufficiently standardized to be recommended for routine practice [7, 21, 22].

In rare cases, suspected NSAID hypersensitivity may be simulated by mastocytosis or mast cell activation syndrome [24]. In such cases, screening by measuring serum basal tryptase may be useful, particularly if the patient has a history of severe reactions.

## Management

Management should consider individual patient factors. In the rare cases of true drug allergy (SNIDR/SNIUA), cross-reactions are not to be expected (Fig. 3), and the approach is usually limited to issuing an allergy passport and avoiding the trigger. On the whole, other NSAIDs can continue to be used.

## Avoidance and an allergy passport

The diagnosis should always be communicated to the patient in written form. Issuing an allergy passport is a common, well established approach in German-speaking countries [25]. It makes the most important management strategy easier: avoidance of the elucidating trigger. As a minimum requirement, the allergy passport should include the generic name of the trigger, together with the dose and the reaction pattern in generally understandable medical language, as well as the correct allergological classification (e.g., NSAID hypersensitivity/NECD (symptom: urticaria); type IV allergy to diclofenac [symptom: maculopapular drug eruption] and how the diagnosis was established [e.g., “confirmed by provocation testing; confirmed by unequivocal patient history”]). The ENDA/EAACI publication includes a practical and extremely useful English form for this purpose [25]. It is also essential that the date and issuer are easily identifiable on the allergy passport in the case of potential medical queries later on.

In the usual case of NERD/NECD/NIUA, cross-intolerance should be mentioned—to the extent that this is known—due to the identical mechanism of action, e.g. “Trigger, acetylsalicylic acid 500 mg, known cross-reactions to all other NSAID.”

## Safe alternative drugs

The allergist needs to assess whether safe alternative drugs are needed. In principle, opiates can be recommended as safe alternatives for analgesia even without testing, since their mode of action, i.e., blockade of the µ‑opioid receptor in the nervous system, is completely different to that of NSAID.

Although marked cross-reactions are to be expected, some patients tolerate NSAID that tend towards stronger COX-2 inhibition, such as oxicams, the COX-2 inhibitors that continue to be available, or, as an alternative, paracetamol. If the patient reports good tolerance of one of these substances in their patient history, this tolerance can be noted in the allergy passport. Should the patient have high analgesic requirements, tolerance of alternative drugs should be demonstrated in a clinically relevant dose. It is important that the tolerated dose ensures sufficient analgesia. For example, good tolerance of the weak analgesic paracetamol at a dose of 250 mg is of no benefit in clinical practice if at least 1000–1500 mg is required for a good analgesic effect. This needs to be taken into consideration in provocation testing.

### Miscellaneous

In specific cases, ASA desensitization can be aimed for, e.g., as recurrence prevention in nasal polyps [17] or anticoagulation in the case of cardiovascular indications where lower maintenance doses with a maximum of 100 mg ASA are adequate [26]. This is started with an extremely small single dose of ASA (generally 1–10 mg), which is then rapidly increased up to the maintenance dose. In the respective, short refractory phase of around 30 min, the next single dose is administered, which explains tolerance and may exceed the initial triggering dose. It is important that the achieved maintenance dose be continued daily and that a maximum pause of only 48 h is permitted, otherwise the desensitization effect is lost. Interestingly, there is often cross-tolerance to other NSAID during this period. The gastrointestinal side effects of long-term ASA use represent a limiting factor in ASA desensitization (note: gastrointestinal side effects such as ulcer, bleeding, and diarrhea are type A side effects; see introduction); since these cause considerable suffering on the patient’s part, there needs be a strict indication for interaction with other disciplines, such as cardiology, neurology, or ENT, in order to consider desensitization.

Another simpler and more pragmatic approach has recently been proposed. Similar to antihistamine premedication in allergen-specific immunotherapy, the tolerated doses of NSAID in NECD and NIUA patients in a small case series of 5 mg desloratadine were significantly increased [27]. However, this highly practical approach still needs to be confirmed in further investigations.

## Abbreviations

ADR: Adverse drug reaction
AERD: Aspirin-exacerbated respiratory disease
AGEP: Acute generalized exanthematous pustulosis
AMG: Medicinal Products Act (*Arzneimittelgesetz*)
ASA: Acetylsalicylic acid
BAT: Basophil activation test
COX: Cyclooxygenase
DRESS: Drug reaction with eosinophilia and systemic symptoms
EAACI: European Academy of Allergy and Clinical Immunology
ENDA: European Network on Drug Allergy
LTT: Lymphocyte transformation test
NECD: NSAID exacerbated cutaneous disease
NERD: NSAID exacerbated respiratory disease
NIUA: NSAID-induced urticaria/angioedema
NSAID: Non-steroidal anti-inflammatory drug
SJS: Stevens–Johnson syndrome
SNIDR: Single NSAID-induced delayed reaction
SNIUAA: Single NSAID-induced urticaria/angioedema or anaphylaxis
TEN: Toxic epidermal necrolysis
